# Review: The Emerging Role of Neutrophil Extracellular Traps in Sepsis and Sepsis-Associated Thrombosis

**DOI:** 10.3389/fcimb.2021.653228

**Published:** 2021-03-17

**Authors:** Zhaoyuan Chen, Hao Zhang, Mengdi Qu, Ke Nan, Hanzhong Cao, Juan P. Cata, Wankun Chen, Changhong Miao

**Affiliations:** ^1^ Department of Anesthesiology, Zhongshan Hospital, Fudan University, Shanghai, China; ^2^ Cancer Center, Zhongshan Hospital, Fudan University, Shanghai, China; ^3^ Anesthesiology and Surgical Oncology Research Group, Department of Anesthesiology and Perioperative Medicine, Nantong, China; ^4^ Department of Anesthesiology and Perioperative Medicine, Anesthesiology and Surgical Oncology Research Group, The University of Texas MD Anderson Cancer Center, Houston, TX, United States; ^5^ Zhangjiang Institute, Fudan University, Shanghai, China

**Keywords:** sepsis, thrombosis, neutrophil extracellular traps, platelets, complement

## Abstract

Patients with sepsis commonly suffer from coagulation dysfunction and lead to the formation of thrombus. During the development of sepsis, neutrophils migrate from the circulating blood to infected tissues and mediate the formation of neutrophil extracellular traps (NETs) that kill pathogens. However, the overactivation of neutrophils can promote the formation of immunothrombosis and even cause disseminated intravascular coagulation (DIC), which damages microcirculation. The outcome of sepsis depends on early recognition and intervention, so clinical evaluation of NETs function may be a valuable biomarker for early diagnosis of sepsis. The interaction of NETs with platelets, complement, and endothelium mediates the formation of immunothrombosis in sepsis. Inhibiting the formation of NETs is also considered to be one of the potential treatments for sepsis. In this review, we will discuss the key role of neutrophils and NETs in sepsis and septic thrombosis, in order to reveal new mechanisms for thrombosis treatment of sepsis.

## Introduction

Sepsis is a life-threatening organ dysfunction caused by a disordered response to infection (Singer et al., 2016). In 2017, the incidence of global sepsis was estimated to be 48.9 million, of which the death toll related to sepsis was as high as 11 million (Rudd et al., 2020). In early sepsis, multiple pathophysiological processes exist simultaneously, such as inflammation and coagulation activation (Cecconi et al., 2018). The coagulation cascade is a complex process, and changes in patients’ coagulation function during sepsis is a predictor of poor prognosis (Gotts and Matthay, 2016). The host will activate blood clotting in the process of defending against infectious pathogens. However, an excessive activation of the coagulation system along with vascular endothelial damage will cause the formation of microvascular thrombus and lead to multiorgan dysfunction (Gando, 2010). An hyperactivated and dysfunctional coagulation system will also manifest as consumption of anticoagulant factors, inhibition of the fibrinolytic system, significant shortening of clot formation kinetics (CFT), and increase clot firmness (CF) (Adamik et al., 2017). As a pro-inflammatory mediator, thrombin, a critical mediator of the coagulation cascade in sepsis, can activate receptors through specific proteases to stimulate monocytes and endothelial cells (ECs).

As the most abundant white blood cell group in humans, neutrophils play an essential role in the body’s innate immunity caused by infection. An excessive activation of neutrophils can lead to the development of multiple organ dysfunction syndromes (Botha et al., 1995). In sepsis, pathogen-associated molecular patterns (PAMPs) or damage-associated molecular patterns (DAMPs) can activate pattern recognition receptors (PRRs) located in neutrophils (Kolaczkowska and Kubes, 2013). In these cells, those molecules will stimulate cell migration from circulating blood to infected tissues. Then, neutrophils will release intracellular granular components, including myeloperoxidase (MPO), neutrophil elastase (NE), and cathepsin G. Activated neutrophils will also release nuclear DNA and form a network structure containing nucleus, cytoplasm, and granular protein, also known as the neutrophil extracellular traps (NETs) (Brinkmann, 2004). NETs mediate the killing process of bacteria, viruses (Narasaraju et al., 2011; Saitoh et al., 2012) and fungi (Urban et al., 2006), however, their dysfunction will result in inability to fight infection, which is evidenced in patients with severe COVID-19 (Middleton et al., 2020). Excessive NETs formation can damage the microcirculation, promote immunothrombosis and lead to diffuse intravascular coagulation as they facilitate the formation of thrombus as a scaffold for thrombosis (Thomas et al., 2012).

In summary, NETs can eliminate pathogens by mediating neutrophils, but their hyperfunction will also cause tissue damage (Kaplan and Radic, 2012). In this review, we will discuss the key role of neutrophils and NETs in sepsis and sepsis thrombosis, to reveal new mechanisms for sepsis treatment.

## The Formation of NETs

In 1971, Lerner first discovered the important role of neutrophils in the process of thrombosis (Lerner R. G. 1971). But it was not until 2004 when Brinkmann and others suggested the concept of NETs (Brinkmann, 2004).

Neutrophils can activate NADPH oxidase through protein kinase C (PKC) and Raf-MEK-ERK signaling pathways to produce reactive oxygen species (ROS). ROS-activated peptidylarginine deiminase 4 (PAD4) modifies specific arginine residues on histones H3 and H4 (Wang et al., 2004) to make linker histone H1 and heterochromatin protein 1b dissociates from the nucleosome structure (Leshner et al., 2012), which mediates the dedensification of chromatin. MPO, NE, and cathepsin G promote further depolymerization of chromatin, thereby destroying the nuclear membrane (Clark et al., 2007). The chromatin is released into the cytoplasm as DAMP to further activate the immune response and form NETs. Treatment with DNase can significantly destroy the structure of NETs (Dąbrowska et al., 2016). The formation of NETs often leads to death of neutrophil, and this process is called NETosis (Vorobjeva and Chernyak, 2020). NETs can be formed in various parts of the body, and NETs can be released wherever neutrophils exist. Therefore, when sepsis occurs, neutrophils are present in different organs, such as the lung, liver, intestine, kidney, heart, and other organs, as well as in the blood circulation throughout the body. And the interaction of platelets, endothelial cells and complement can mediate the formation of NETs and immunothrombosis.

## Markers of NETs

The outcome of sepsis depends on early diagnosis and intervention. Thus, it has been suggested that early recognition of NETs may be a valuable biomarker of early sepsis. The degree of NETs formation is significantly related to the severity of the disease, and can independently predict the development of diffuse intravascular coagulation (DIC) and mortality (Abrams et al., 2019). Cell free DNA (cfDNA), NETs related MPO-DNA complex, citrullinated-histone H3-DNA (cit-H3-DNA) complex and deoxyribonuclease I (DNase I) can indirectly reflect NETs formation in the blood of patients with sepsis (Wang et al., 2016). For instance, cit-H3-DNA is also associated with NETs in patients with severe respiratory infections (Hirose et al., 2014). Also, DNA release, elastase production and inflammatory cytokines can affect the severity and organ failure of patients with sepsis (Kumar et al., 2019).

These markers have the potential to detect NETs, although they are not directly achieved by detecting NETs. At the same time, some markers are not only related to NETs, but also related to infections caused by other mechanisms. For example, the concentration of cfDNA in plasma can activate the nucleic acid sensing pathway to generate inflammation, and participate in the formation of sepsis (Sandquist and Wong, 2014). In addition, the circulating concentration of some markers is unstable, lacking detection sensitivity and specificity.

Abrams et al. (2019) found that co-incubation of neutrophils with the plasma or serum of patients with sepsis can directly induce the production of NETs. Compared with healthy volunteers, the plasma of patients with sepsis can cause a large number of NETs, and the degree of plasma NETs in patients with thrombocytopenia, abnormal prothrombin time, activated partial thromboplastin time, fibrinogen, and D-dimer are closely related. Therefore, these indicators can also be used as indirect means to reflex NETs (Abrams et al., 2019; Kumar et al., 2019). Correctly detecting the formation of NETs is essential for identifying early sepsis.

## Factors in NETs-Associated Thrombosis

### Vascular Endothelium

The integrity of endothelial structure and function is the key to maintaining vascular homeostasis. Endothelial injury plays an important role in thrombosis. In the development of sepsis, neutrophils are recruited to the site of inflammation through rolling, activation, firm attachment, and extravasation on ECs (Ley et al., 2007). The rolling of neutrophils on activated ECs is caused by the action of P-selectin and P-selectin glycoprotein ligand 1 (PSGL-1). By activating integrin αLβ2 (LFA-1) and binding to intercellular adhesion molecule 1 (ICAM-1), P-selectin participates in the further rolling and firm adhesion of neutrophils. Through the interaction between CXC chemokine ligand 1 (CXCL1) and its receptor CXC chemokine 2 (CXCR2), neutrophils are recruited to the site of inflammation (Grover and Mackman, 2018).

In septic-infected mice, the release of extracellular histones led to infiltration of neutrophils, vacuolation of ECs, intra-alveolar hemorrhage, and vascular thrombosis (Xu et al., 2009). When ICAM-1 inhibitors inhibited the homing of neutrophils to ECs, thrombosis in mice was reduced. NETs promote the activation of ECs and increase thrombosis through the synergistic effect of IL-1α and cathepsin G (Folco et al., 2018). Matrix metalloproteinases-9 (MMPs-9) in NETs can activate MMP-2 of ECs and cause endothelial dysfunction (Carmona-Rivera et al., 2015). NETs can induce ECs to release adhesion factors and tissue factor (TF), further recruit inflammatory cells, and promote thrombosis (Rabinovitch, 2016). In sepsis, lipopolysaccharide (LPS) induces the activation of peptidylarginine deiminase (PAD) and the formation of NETs mediated by the PAD-NET-CitH3 pathway, which changes the permeability of the pulmonary vascular endothelium (Liang et al., 2018). The induction of ECs by NETs also has two sides. Low concentrations of NETs can promote the release of inflammatory factors from ECs through the TLR4/NF-κB signaling pathway (Aldabbous et al., 2016), and NETs can also induce death of ECs in a dose-dependent manner (Saffarzadeh et al., 2012). NETs can amplify endothelial dysfunction associated with thrombosis. Vascular endothelium plays an important role in the occurrence and development of thrombus in sepsis.

### Platelets

Platelets play an essential role in coagulation (van der Meijden and Heemskerk, 2019). Platelets can recruit leukocytes to the site of infection in the early stages of sepsis. Platelet activation induced by pathogens and platelet agonists, such as thrombin, arachidonic acid, collagen, or ADP can mediate the development of procoagulant, prothrombotic and inflammation, and ultimately lead to vascular endothelium damage. Activated platelets can also roll and adhere to the surface of neutrophils to form aggregates and enhance their ability to kill pathogens (Jenne et al., 2013; Jenne and Kubes, 2015). NETs further promote platelet adhesion, activation, aggregation, and exert procoagulant activity, thereby providing a scaffold for thrombosis (Fuchs et al., 2010), forming positive feedback activation of coagulation. NETs can also recruit red blood cells, promote von Willebrand Factor (VWF), fibrinogen and fibrin deposition, thereby inducing venous thrombosis (Fuchs et al., 2010).

Sepsis is usually accompanied by thrombocytopenia and occlusion of small blood vessels, called disseminated intravascular coagulation (DIC). The substances produced by NETs can either diffuse throughout the interstitium of organs, or be released into the vascular lumen and attach to the vascular wall of narrow capillaries (Carestia et al., 2016). In the mouse model of pneumonia caused by sepsis, thrombocytopenia usually worsened sepsis, led to increased bacterial growth in the blood and lungs, and reduced the survival rate of animals (de Stoppelaar et al., 2014).

LPS can initiate the non-classical activation of platelets, and activate TLR4 on platelets which will also lead to the release of the NETs (Carestia et al., 2016). The activation of platelets by LPS can induce platelet-dependent tissue factor pro-coagulant activity (TF-PCA) and promote the production of thrombin in a TLR4-dependent manner (Schattner, 2019). As one of the substances released by NETs, histones can induce the production of thrombin (Fuchs et al., 2011).

**Figure 1:**
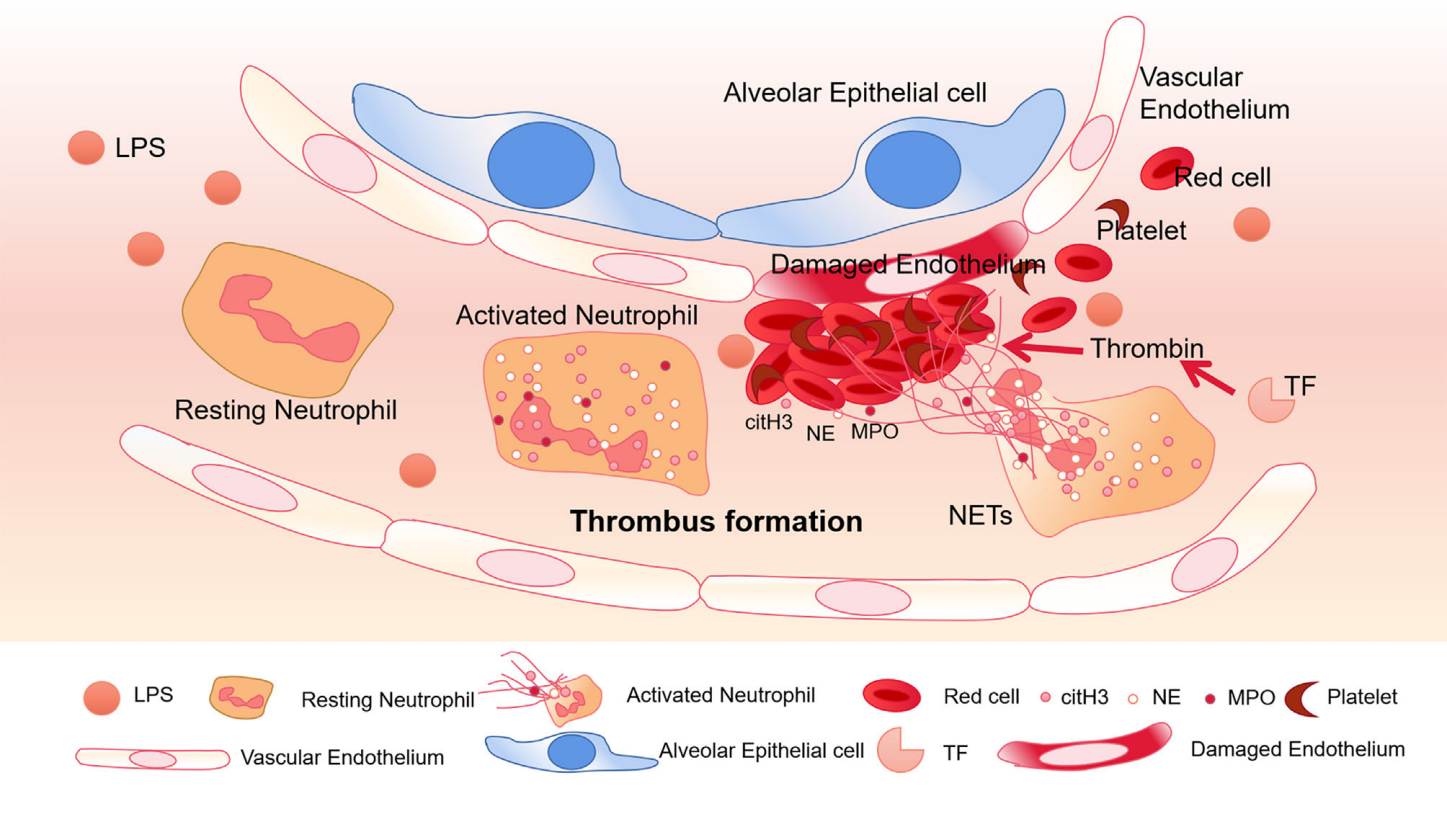
Thrombus formation in platelets associated with NETs. In pneumonia of sepsis, LPS released by gram-negative bacteria can activate platelets. Neutrophils migrate into pulmonary capillaries, and interact with circulating platelets to form aggregates. Neutrophils release NETs, and citH3, NE, and MPO adhere to the NETs. Under the action of tissue factor (TF), NETs form thrombus together with red blood cells and platelets, destroying vascular ECs.

To a certain extent, NETs can trigger pro-thrombotic and pro-coagulant platelet-mediated responses by interacting with TLR4. As a key receptor, TLR4 on platelets may be an important factor in septic DIC (Semeraro et al., 2011). In septic mice, neutrophils adhere to the endothelial cells of the lung and liver sinusoids, and platelets activated by LPS can stick to neutrophils to promote the production of NETs (Clark et al., 2007). The α-toxin produced by Staphylococcus aureus can bind to A disintegrin and metalloprotease 10 (ADAM10), which also causes the activation of platelets and neutrophils, and the formation of platelet-neutrophil aggregates (Powers et al., 2015). In addition, intravenous injection of α-toxin can rapidly accumulate platelets and form thrombus in the liver sinusoids and glomeruli through GPIb, leading to multiple organ dysfunction (Surewaard et al., 2018). In COVID-19 patients, it was also found that the expression of P-selectin increased, and the aggregation of platelets-neutrophils and monocytes increased (Bhanu Kanth Manne, 2020) which was associated to thrombotic complications. Therefore, platelets play an important role in abnormal blood coagulation caused by sepsis (**Figure 1**).

### Complement

In recent years, it has been discovered that NETs can activate three different complement pathways to form thrombosis. The complement system is stimulated by active coagulation factors, causing the immune system to recruit neutrophils (Berends et al., 2014). The complement system is composed of serine-protease cascade reactions, including the continuous cleavage of complement proteins, which ultimately leads to the formation of membrane attack complex (MAC) (Morgan, 2016).

**Figure 2:**
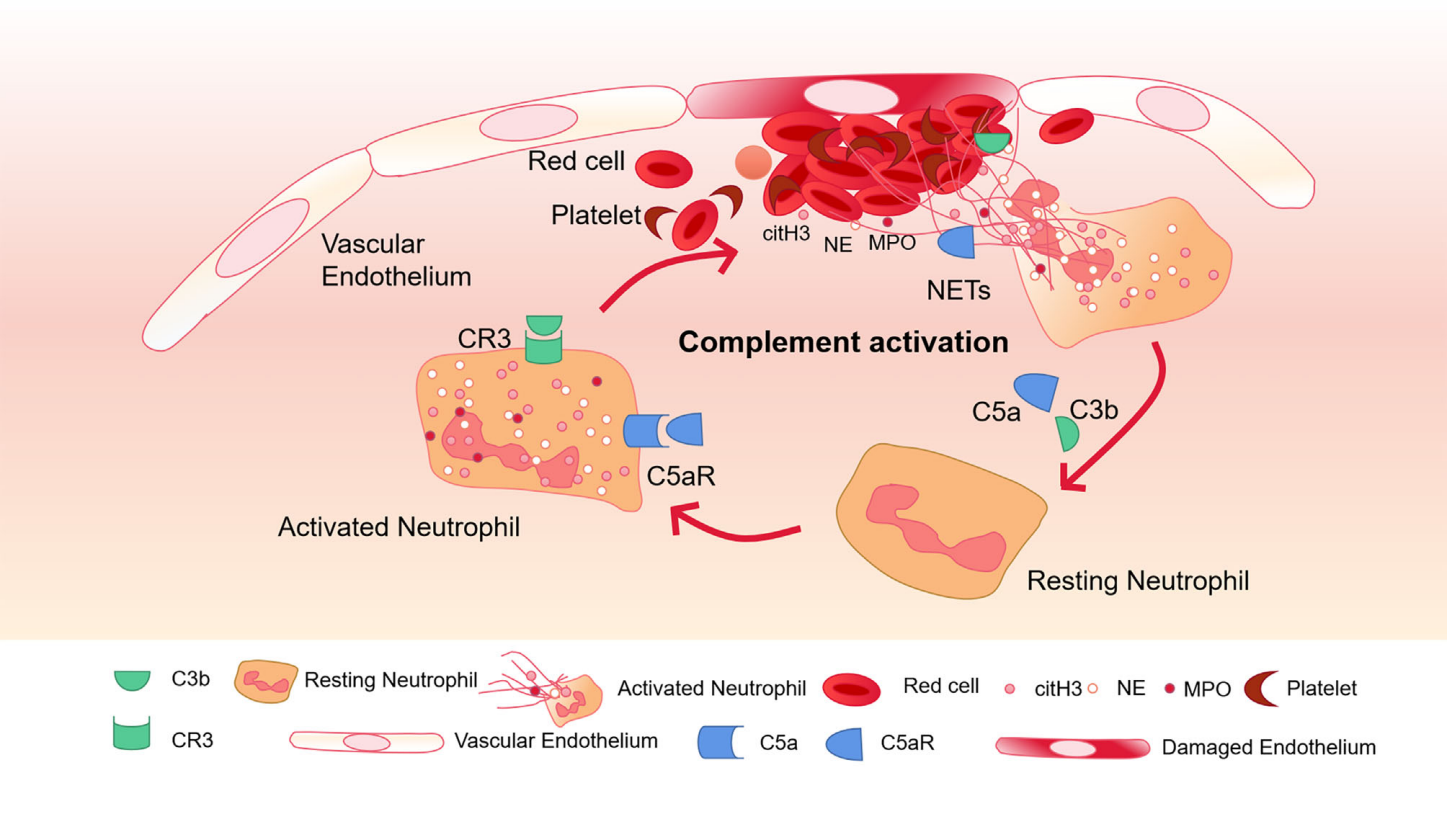
Complement activation and NETs-mediated thrombosis. Complement C5a and C3b activate neutrophils and bind to CR3 and C5aR receptors on the surface of neutrophils to further promote the release of NETs. The activation of neutrophils can lead to endothelial cell damage, activation of the coagulation system, and further activation of complement through NETs, and finally amplify the inflammatory response.

Complement C3 is an important mediator in the complement system and activates neutrophils during sepsis (Yuen et al., 2016). Neutrophils from C3a receptor (C3aR) -deficient mice cannot form NETs. When C3 containing serum from healthy mice is exogenously administered, their function is restored. This shows that complement is closely related to the formation of NETs (Yipp et al., 2012). Blocking the complement receptor 3 (CR3) that binds to opsonin iC3b can inhibit NETosis (Behnen et al., 2014). C3b, as an important upstream component to generate MAC, has also been found in NETs (Behnen et al., 2014). On PMA-induced NETs, Factor B and C3 deposition have been found, and the combination of C1q and NETs can prevent itself from being degraded by DNase I (Papayannopoulos, 2018). Thereby maintaining the structure of NETs (Elkon and Santer, 2012; Leffler et al., 2012). Complement C5a can recruit and activate neutrophils, leading to the up-regulation of immune receptors such as Toll-like receptors (TLR) and complement receptors (Wang et al., 2015). In addition, using C5a to pre-stimulate neutrophils enhanced their ability to generate NETs (Wang et al., 2015). The complement molecules in the blood can be deposited on NETs and continue to function. Properdin, Factor B and C3 have all been found to exist on NETs and exert their blood coagulation and antibacterial functions (Yousefi et al., 2009) (**Figure 2**).

### Immunothrombosis

In recent years, researchers have proposed that there may be an effect mechanism in the link between blood coagulation and innate immune response that mediates the formation of immunothrombosis. Initially, NETs were found in patients’ arterial or venous thrombosis. Researchers gradually realized that NETs provide a scaffold to recruit red blood cells, platelets, white blood cells, as well as bind plasma proteins. The aggregation of NETs in the coronary arteries suggested that NETs might contribute to the growth and stability of thrombus (de Boer et al., 2013). By analyzing ischemic stroke thrombosis, DNA, MPO and citrullinated histone H4 were found to be positive for triple co-staining, and NETs were abundant in different types of stroke thrombosis (Ducroux et al., 2018). Infection accelerates neutrophils recruitment and activation, leading to a more pronounced involvement of NETs in thrombosis. In patients with abdominal aortic aneurysm infected with Porphyromonas gingivalis, the recruitment of neutrophils and the formation of NETs were increased, and wound healing was inhibited (Delbosc et al., 2011). Infection-driven NETs formation also leads to pulmonary thrombosis associated with sepsis (Jimenez-Alcazar et al., 2017). Because NETs can stimulate fiber remodeling, NETs may promote the development of venous thrombosis (Chrysanthopoulou et al., 2014). NETs may also be a prethrombotic lesion and play a key role in thrombosis.

The coagulation system is activated by pathogens and helps the host defend against infection by reducing the spread of pathogens and killing them (Engelmann and Massberg, 2013). Immunothrombosis can mediate the identification of pathogens and damaged cells, and inhibit the spread and survival of pathogens. This phenomenon also exists in sepsis. Neutrophils promote the capture and degradation of pathogens through the NETosis pathway, and the decondensed chromatin and antibacterial proteins are released from neutrophils to form NETs. Through the release of NETs, neutrophils participate in the formation of immunothrombosis. Intravascular coagulation induced by NETs resulted in extensive microvascular occlusion and multiple organ failure in a variety of sepsis mouse models (McDonald et al., 2017).

The presence of senescent and pro-inflammatory neutrophils in the circulation of miR-146a-deficient mice leads to increased thrombosis in the LPS-induced sepsis model (Arroyo et al., 2020). Exposure of the endothelium to pathogens and PAMP triggers a pre-adhesion phenotype that leads to neutrophil binding. NF-κB signal is involved in the circulatory process of thrombus and inflammation (Mussbacher et al., 2019). Neutrophils can secrete molecules that promote the initiation of the coagulation cascade (Darbousset et al., 2012), and TF plays an important role in this process (Smadja, 2014).

The two-way effect of NETs and platelets represents the connection between these phenomena. Whether in infectious or non-infectious diseases, the interaction of platelets and neutrophils is crucial (McDonald et al., 2017), and constitutes a vicious circle in the process of immunothrombosis Therefore, immunothrombosis may be an important physiological process of intravascular immunity, and its imbalance may be one of the potential events that trigger thrombotic diseases and lead to intravascular coagulation in sepsis.

## Potential Therapeutic Targets

Although NETs are increasingly recognized as important therapeutic targets, there is currently no clear drug that regulates NETs to guide the treatment of sepsis and sepsis-related thrombosis. Several potential targets of NETs are listed below.

### TLR4 Blocker

The downstream reaction caused by the combination of TLR4 and LPS is one of the classic pathways of sepsis. In addition, TLR4 on platelets is also involved in the induction of NETs in mice and humans (Clark et al., 2007). TLR4^-/-^ septic mice did not enhance the thrombotic response, and circulating ICAM-1 was significantly reduced (Obi et al., 2017). Inhibition of TLR4 can improve the prognosis of sepsis (Schattner, 2019), so TLR4 blockers may be used as a potential new method for the treatment of sepsis. 2-acetamidopyranoside compound (MW 389) acts as a TLR blocker to inhibit the production of TNF-α and iNOS RNA in a mouse model of LPS-induced sepsis (Neal et al., 2013). In addition, statins, and angiotensin receptor blockers (ARBs) have been found to have inhibitory activity on the TLR4 signaling pathway. Fluvastatin, simvastatin and atorvastatin showed effective inhibition of the TLR4 pathway (Gao et al., 2017). Candesartan can inhibit the activation of TLR4 induced by LPS (Dasu et al., 2009), and valsartan of the angiotensin-receptor blocker family (Yang et al., 2009) has been shown to reduce the release of pro-inflammatory cytokines by inhibiting TLR4 signaling. TAK-242 (resatorvid) is a small molecule specific inhibitor of TLR4. By binding to the intracellular domain of TLR4, it disrupts the interaction between TLR4 and adaptor molecules, thereby inhibiting TLR4 signal transduction and preventing the production of inflammatory mediators induced by LPS (Matsunaga et al., 2011). However, in large-scale clinical trials, TAK-242 failed to suppress cytokine levels in patients with sepsis, shock, or respiratory failure (Rice et al., 2010). Even if drugs are given in the early stages of sepsis, when sepsis is diagnosed, there is already an inflammatory response that cannot be suppressed. Therefore, it may be more important to recognize the signs of sepsis and prevent them in time.

As a ligand of LPS, TLR4 can prevent the cascade of inflammation by intervening in the early stage of sepsis, but its complete blockade may also lose the body’s innate immune response to endotoxin. Therefore, it is still necessary to consider the pros and cons of its use before putting it into clinical use.

### PAD4 Inhibitors

Overexpression of peptidyl arginine deiminase 4 (PAD4) leads to the elimination of chromatin and the release of NETs in cells *in vitro*. PAD4 causes the loss of the positive charge on the arginine guanidine group and unfolds the DNA strand to form NETs. PAD4 plays an important role in the formation of NETs (Franck et al., 2018). Therefore, the activation of PAD4 may be one of the targets of NETs formation. In a sepsis model, PAD4 knockout mice showed improved survival rate, reduced severity of organ dysfunction, and lower development of sepsis (Biron et al., 2018). PAD4 inhibitor BB-Cl-amidine can protect NETs-mediated vascular injury and endothelial dysfunction in mouse models (Knight et al., 2015). Perdomo et al. found that thrombocytopenia and thrombosis are two separable processes. Even in the absence of NETs, heparin may also lead to the reduction of platelets. Knockout of PAD4 or use of PAD4 inhibitor GSK484 will inhibit heparin-induced thrombocytopenia/thrombosis (HIT) thrombocytopenia and thrombosis (Perdomo et al., 2019). PAD4 inhibitor GSK484 can also affect kindlin-3 in mouse neutrophils and inhibit thrombosis caused by NETs (Yan et al., 2019). The PAD4 inhibitor Cl-amidine affects endothelial function and prethrombotic phenotype by blocking NETs (Knight et al., 2013), and can also inhibit tissue damage associated with inflammatory bowel disease mouse models (Chumanevich et al., 2011). Therefore, PAD4 may be one of the potential targets to inhibit the formation of NETs, which can interfere with thrombosis caused by inflammation without affecting the coagulation system.

### Heparin

The role of heparin therapy in anticoagulation is unquestionable. However, the treatment of thrombus caused by heparin in NETs is still controversial. Low-dose heparin (250U/kg) treatment can reduce the expression of NETs, histones, and pro-inflammatory factors (Sun et al., 2020). A large amount of heparin will produce antibodies against platelet factor 4 (PF4) and heparin complexes, leading to excessive activation of coagulation to activate platelets and promote thrombosis (Pouplard et al., 1999). In addition, heparin can directly induce the formation of NETs, but NETs cannot be induced by low molecular weight heparin, heparin analogs or heparin sulfate [low molecular weight heparin(LMWH), heparin mimetics or heparan sulfate] (Lelliott et al., 2020). However, there are also reports in the literature that heparin and heparin-derived drugs (including unfractionated heparin, LMWH and occasionally fondaparinux) can induce immune responses, leading to HIT (Perdomo et al., 2019). This suggests that we need to pay attention to the dosage when using heparin in clinical practice, or use heparin substitutes for anticoagulation therapy to avoid activating NETs.

### Other Treatments

In septic patients, the concentration of IL-8 is closely related to the formation of NETs. IL-8 activation of mitogen-activated protein kinase is one of the main pathways for the formation of NETs in patients. Inhibition of IL-8 can significantly reduce NETosis (Abrams et al., 2019). Histones can activate neutrophils to form a network, and recombinant thrombomodulin (rTM) can inhibit the formation of NETs induced by histones (Binita Shrestha, 2019).

There is a dynamic platelet thrombin axis in sepsis, which promotes intravascular coagulation and microvascular dysfunction. DNase can inhibit the formation of NETs by degrading DNA. DNase1 and DNase1-like 3 can be expressed independently, degrade NETs in sterile neutrophilemia and sepsis, provide double protection, and protect the host from the harmful effects of intravascular NETs (Jimenez-Alcazar et al., 2017). Elimination of NETs by infusion of DNase, or in mice deficient in NETs formation caused by PAD4 deficiency, can be found to significantly reduce thrombin activity, platelet aggregation and improve microvascular perfusion (McDonald et al., 2017). A summary of potential targets for NETs therapy is shown in **Table 1**.

**Table 1:** Potential targets for NETs therapy.

Mechanism	Drug	References
TLR4 blocking agent	MW 389	(Neal et al., 2013)
	Statins (Fluvastatin, Simvastatin, Atorvastatin)	(Gao et al., 2017)
	ARBs (Candesartan, Valsartan)	(Dasu et al., 2009; Yang et al., 2009)
	TAK-242	(Matsunaga et al., 2011)
PAD4 inhibitor	BB-Cl-amidine	(Knight et al., 2015)
	GSK484	(Perdomo et al., 2019)
	Cl-amidine	(Knight et al., 2013)
Heparin	Low-dose heparin	(Sun et al., 2020)
Anti–IL-8	Anti–IL-8 blocking mAb	(Abrams et al., 2019)
Prevent histone-induced NETs formation	Recombinant thrombomodulin	(Binita Shrestha, 2019)
Degrade DNA in NETs	DNase1	(Jimenez-Alcazar et al., 2017)
	DNase1-like 3	(Jimenez-Alcazar et al., 2017)

## Conclusion

NETs have been confirmed to be related to inflammation, infection, and autoimmune diseases, and play an important role in sepsis and thrombosis caused by sepsis. NETs can not only play a role in immune defense in the inflammatory response, on the other hand, their excessive activation can also lead to a strong immune response and cause adverse consequences. In sepsis, NETs mainly through the interaction of neutrophils and platelets, leading to the functional damage of vascular ECs and the formation of immunothrombosis, further promoting the occurrence of microcirculation disorders.

The existence of NETs suggests that in the treatment of sepsis, whether a new method can be used to avoid excessive activation of inflammation and prevent the further occurrence of sepsis without affecting blood coagulation. By detecting the formation potential of NETs in the peripheral circulation, it can be used clinically to identify patients at risk of DIC and poor prognosis of sepsis. Based on the evidence that NETs and platelets interact to promote intravascular coagulation and its diffusion, we believe that the early recognition of NETs in sepsis can prompt early treatment of sepsis and DIC high-risk patients, and also provide a new direction for its treatment.

## Author Contributions

ZC, HZ, WC, and CM provided the conception and the structure of the article. ZC wrote the draft. All authors contributed to the article and approved the submitted version.

## Funding

This research was supported by the National Key Research and Development Program of China (NO. 2020YFC2008400), the National Natural Science Foundation of China (NO.81873948, 81871591), Clinical Research Plan of SHDC (NO. SHDC2020CR4064, SHDC2020CR1005A), Shanghai Shenkang hospital development center, clinical science and technology innovation project (NO. SHDC12018105), 2019 Fudan University Zhuo-Xue Project (NO. JIF159607); Shanghai Sailing Program (No. 20YF1418400); Shanghai Leading Talent (NO: 2019-112).

## Conflict of Interest

The authors declare that the research was conducted in the absence of any commercial or financial relationships that could be construed as a potential conflict of interest.
